# Secondhand homes: The multilayered influence of woodpeckers as ecosystem engineers

**DOI:** 10.1002/ece3.7932

**Published:** 2021-07-22

**Authors:** Faith O. Hardin, Samantha Leivers, Jacquelyn K. Grace, Zachary Hancock, Tyler Campbell, Brian Pierce, Michael L. Morrison

**Affiliations:** ^1^ Department of Rangeland, Wildlife, and Fisheries Management Texas A&M University College Station TX USA; ^2^ Natural Resources Institute Texas A&M University College Station TX USA; ^3^ Department of Ecology & Conservation Biology Texas A&M University College Station, Texas TX USA; ^4^ Department of Integrative Biology Michigan State University East Lansing MI USA; ^5^ East Foundation San Antonio TX USA

**Keywords:** ecosystem engineers, insect communities, nesting success, secondary cavity nesters, species interactions, woodpeckers

## Abstract

Ecosystem engineers alter, and can be influenced in turn by, the ecosystems they live in. Woodpeckers choose foraging and nesting sites based, in part, on food availability. Once abandoned, these cavities, particularly within areas of high forage, may be crucial to secondary cavity‐nesting birds otherwise limited by cavities formed through decay. Our study examined factors that influence the nesting success of primary cavity nesters and the subsequent impact on secondary cavity‐nesting birds. Using 5 years of point count data, we monitored the outcomes of cavity‐nesting birds in South Texas. We used logistic‐exposure models to predict daily survival rates based on cavity metrics and used woodpecker foraging trends and insect surveys to determine if nesting where woodpeckers actively forage benefits secondary cavity‐nesting birds. Both woodpeckers and secondary cavity nesters shared predictors of daily survival; nests were more successful in cavities with small openings in minimally decayed trees. All secondary cavity nesters had higher probabilities of success when nesting in an abandoned woodpecker cavity, opposed to ones formed by decay. Woodpeckers tended to forage in areas with higher‐than‐average levels of the insect orders Coleoptera, Hymenoptera, and Orthoptera, and secondary cavity nesters had higher rates of success when nesting in these areas. Our results suggest abandoned woodpecker cavities may be constructed in a way that directly benefit secondary cavity nesters. Additionally, we suggest an interplay between these ecosystem engineers, food availability, and secondary cavity nesters: Woodpeckers engineer superior nesting cavities in areas where food is more abundant, and the resultant cavities in areas of high forage may benefit local secondary cavity nesters. Our findings indicate that there is still much to be explored in the role of ecosystem engineers, and how they influence local communities on multiple trophic levels.

## Graphical abstract

Woodpeckers leave behind nesting cavities that are crucial to secondary cavity‐nesting organisms. At a study site in southern Texas, USA, we evaluated how local secondary cavity‐nesting birds may be influenced by the placement and structure of abandoned Golden‐fronted woodpecker (Melanerpes aurifrons) cavities, in terms of both food resources and nesting success. We found that secondary cavity nesters had higher nest success in abandoned woodpecker cavities than in naturally occurring cavities, trends that correlated with both food resources in the area and the survival rates of the woodpecker chicks. Contrary to our previous understanding of woodpecker ecology, woodpeckers in this region chose dense, live trees in which to excavate nests as opposed to soft, decayed trees, potentially influenced by anti‐predator tactics and the extreme heat of the region.

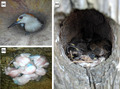

## 1 INTRODUCTION

Understanding complex horizontal (i.e., within a single trophic level) and vertical diversity (i.e., food web interactions) can allow for a deeper understanding of multitrophic interactions (Duffy et al., 2007). Ecosystem engineers interact with multiple trophic levels by directly impacting not only ecological associations but also the behavior of animals within an ecosystem (Bangert and Slobodchikoff, 2004; Lill & Marquis, 2003; Rozhkova‐Timina et al., 2018). As such, we often think about how the actions of engineers affect others, but this is inherently influenced by the decisions they make for themselves. As a result, understanding why and where engineers perform certain actions will help us better understand the interconnected layers within these systems.

The woodpecker acts as an ecosystem engineer by creating multiple partially and fully formed cavities each year (Catalina‐Allueva and Martin, 2021; Floyd & Martin, 2016; Loye & Carroll, 1998; Wiebe, 2017) that, once abandoned, are used by a variety of secondary cavity‐nesting species (i.e., species that require a cavity to nest in but cannot create the cavity themselves) (Cockle et al., 2010; Newton, 1994; Pakkala et al., 2019). Depending on the preferences of the woodpecker (live versus dead trees, low versus high vegetation cover, etc.), the cavities left behind may be superior nesting spaces when compared with ones formed by decay (Blanc and Walters, 2008; Pakkala et al., 2019). Secondary cavity‐nesting birds often take advantage of these abandoned cavities; indeed, suitable cavity availability may be a limiting factor for secondary cavity nest bird populations (Segura, 2017; Tiainen et al., 1984; Tarbill et al., 2015), though this effect is highly dependent on environmental factors like forest density and the availability of decay‐formed cavities (Wesołowski, 2007; Wesołowski and Martin, 2018). Additionally, abandoned woodpecker cavities may have increased antipredation benefits (when compared to decay‐formed cavities) because of smaller entrance holes and deeper depths (Mikusiński et al., 2018; Paclík et al., 2009). On the other hand, cavities created in highly decayed wood may repel secondary cavity nesters as soft walls provide less protection against predators (Wesołowski, 2007), and some woodpecker species can act as predators themselves (Husak, 1995; Walankiewicz, 2002; Wesołowski, 2002; Wilson & Walters, 2020).

Woodpecker cavity construction varies across species. For example, some species choose to excavate highly decayed (i.e., soft) wood for ease of excavation, like the northern flicker (*Colaptes auratus*) (Raphael & White, 1984), while others choose more dense live trees, like the red‐cockaded woodpecker (*Dryobates borealis*) (Jackson et al., 1979). Other nest characteristics that have been shown to influence nest success include the diameter of the tree, and the height, diameter, and depth of the cavity within the tree (Li & Martin, 1991; Loye & Carroll, 1998; Mannan et al., 1980; Newlon, 2005). Most woodpecker species, especially those within the genus *Melanerpes,* tend to excavate cavities with small openings, only large enough for their bodies (5–6 cm diameter), and high in trees, presumably to avoid predation (Sedgwick & Knopf, 1990; Paclík et al., 2009; Straus et al., 2011).

Woodpecker resources can be defined both in terms of suitable nesting locations (cavities that provide protection from the elements and from predation) and food availability (Bonnot et al., 2009; Conner et al., 1994; Mikusiński, 2006). These resources have been shown to be directly linked to woodpecker nest site location and the distance they travel during foraging bouts (Conner, 1981; Lorenz et al., 2016; Wiktander et al., 2001). For example, the black‐backed woodpecker (*Picoides arcticus*) selects nesting sites based on infestations of the mountain pine beetles (*Dendroctonus ponderosae*) (Rota et al., 2015), and the European three‐toed woodpecker's (*Picoides tridactylus*) home range size is negatively correlated with the number of trees with suitable diameters for cavity excavation and can dramatically benefit from increases in deadwood after forest fires, though these effects are short lasting (Gustafsson et al., 2019; Pechacek & d'Oleire‐Oltmanns, 2004).

Most woodpeckers have specialized diets, often corresponding with their ability to excavate wood of varying density or competition with other local woodpecker species (Hanson & North, 2008; Torok, 1990). However, some are more omnivorous, like the Golden‐fronted woodpecker (*Melanerpes aurifrons*), whose diet consists heavily of wood‐boring and bark‐dwelling beetles, but also commonly forages for ants (Hymenoptera), grasshoppers (Orthopteta), small vertebrates, cactus fruits, and nuts (Kujawa, 1984; Oberholser and Kincaid, 1974; Schroeder et al., 2013). In terms of food availability, an increase in population size of one insect taxa can sometimes be mirrored by similar increases in other insect taxa, usually correlating with changing environmental conditions and available resources (Brooks et al., 2012; Koivula, 2011; Lehnert et al., 2013). (Mikusiński, 2006). Therefore, woodpeckers and secondary cavities nesters may have an interwoven relationship with food resources as the woodpecker acts as an ecosystem engineer by constructing cavities in areas with increased foraging opportunities for both taxa.

The Golden‐fronted woodpecker (*Melanerpes aurifrons*), hereafter referred to as “woodpecker,” is a poorly studied, medium‐sized bird, whose range extends from Central America to Texas (Schroeder et al., 2013; Wetmore, 1948). Cavities are typically constructed to be deep (31 cm) and to have small openings, approximately 5 cm wide (Skutch, 1969). As with many woodpeckers, it is assumed these dimensions help to reduce predation by making it difficult for the predator to enter and remove chicks (Wesołowski, 2002; Wilcove, 1985). Clutch sizes vary from 4–7 and pairs successfully fledge 1–4 young per year (Skutch, 1969).

**FIGURE 1:**
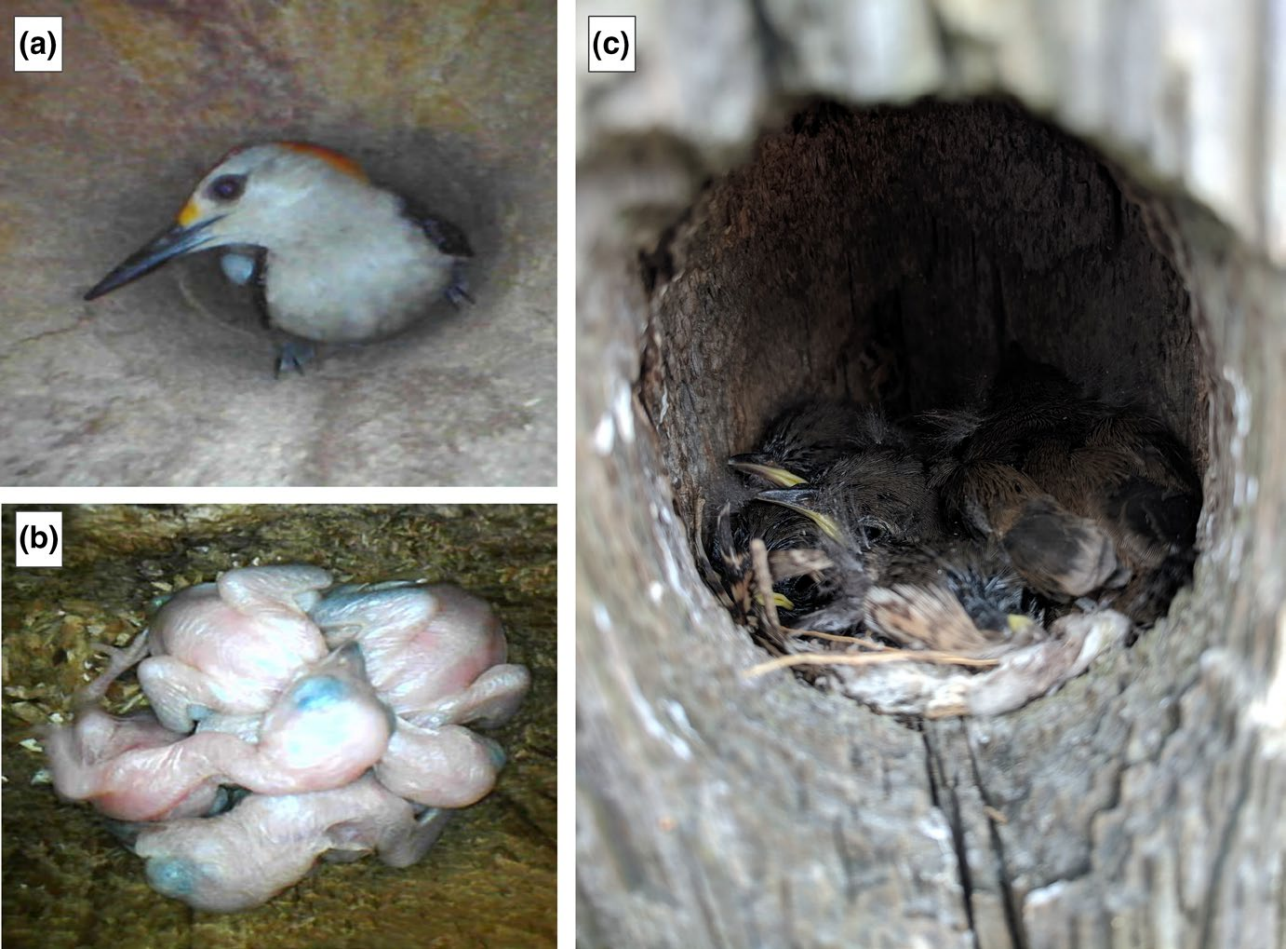
Cavity‐nesting birds in south Texas, images taken with telescoping Bluetooth camera. Photo credit: Faith Hardin. (a) Adult male Golden‐fronted woodpecker with eggs. (b) Golden‐fronted woodpecker chicks, 3 days after hatching. (c) Bewick's wren chicks at one and a half weeks old in abandoned woodpecker cavity

The Golden‐fronted woodpecker (hereafter referred to as woodpecker) is in decline across their Texas distribution and is considered a species of concern in the Texas Wildlife Action Plan (Bender, 2007). As with other woodpecker species, it acts as an ecosystem engineer, providing nesting cavities for secondary cavity‐nesting birds throughout their range (Husak & Maxwell, 1998), see Figure 1. Determining the factors that influence the nest site location and construction of cavities is crucial to not only understand the conservation needs of the woodpecker but also for the conservation and basic ecology of reliant secondary cavity nesters. This is especially true given that changes in the environment (e.g., global warming, woody encroachment) may influence woodpecker behaviors or preferences and therefore have consequences for secondary cavity nesters (Wesołowski et al., 2021).

We conducted an observational study on woodpecker nesting success in relation to nesting site locations, cavity construction, foraging distances, and local insect biomass, along with the nesting success of the four most common secondary cavity‐nesting birds in our study area. These species include the black‐crested titmouse (*Baeolophus atricristatus*), ash‐throated flycatcher (*Myiarchus cinerascens*), brown‐crested flycatcher (*Myiarchus tyrannulus*), and Bewick's wren (*Thryomanes bewickii*), in the southern Texas Tamaulipan Brushlands (Baumgardt et al., 2019).

The objectives of our study were to determine (a) what cavity metrics most likely influence the success of Golden‐fronted woodpecker nests (e.g., live or dead trees, height of cavity), (b) how existing cavity structure directly influences secondary cavity nesters' success, and (c) whether food resources are greater around the areas that woodpeckers and secondary cavity nesters establish nests in. We predicted (a) that woodpeckers would have higher nest success in cavities with strong antipredator characteristics (e.g., higher in trees with deep cavity depths and small openings), (b) that secondary cavity nesters would have higher nest success in abandoned woodpecker cavities, because they would have stronger antipredator characteristics than cavities formed by decay and, (c) there would be a positive correlation between levels of commonly eaten insects and the nesting locations of both the woodpecker and secondary cavity nesters, and that this increase would positively correlate with nesting success.

## 2 MATERIALS AND METHODS

### 2.1 Study area

Our study was conducted on the East Foundation's ~61,000 ha San Antonio Viejo (SAV) ranch located in Jim Hogg and Starr counties, ~25 km south of Hebbronville, Texas. Annual rainfall during the study year (2019) for this region was ~30 cm, and the mean temperature during the breeding season (March−July) was ~27.8°C similar to the 30‐year norm for this region (PRISM Climate Group, 2019). This area is representative of the Tamaulipan/Mezquital Thornscrub ecological region, and the ranch is composed of rolling sand plains and some caliche soils containing black brush (*Acacia rigidula*), drought hardy grasses, southern live oak (*Quercus virginiana*), and the main species of woody plant that grows to a diameter large enough to facility cavity excavation, honey mesquite (*Prosopis glandulosa*). Depending on soil types, the mesquite can form dense forests or be scattered irregularly across the landscape. As such, woodpecker cavities are found almost exclusively in mesquite, and very rarely, live oak. The most common nest predators of the region are tree‐climbing snakes (Davis et al., 2019). The SAV supports approximately 70 residential bird species and 45 migratory species (Baumgardt et al., 2019).

### 2.2 Nest location and monitoring

The East Foundation has an extensive long‐term breeding bird dataset, consisting of point count surveys conducted during the spring and summer months of 2014–2020 (and continuing to date) (Baumgardt et al., 2019). The surveys consisted of 25, 12‐point groups established through stratified randomization by vegetation type and were visited 6 times per month. Each point was 400 m apart, and two observers recorded birds by sight and sound within a 200 m radius. From this dataset, we were able to conclude that the Golden‐fronted woodpecker is by far the most common species of woodpecker within the ranch, with only one other woodpecker observed, the ladder‐backed woodpecker (*Dryobates scalaris*), and this only on average twice a year across all surveys. Furthermore, we never observed a nesting ladder‐backed woodpecker throughout this project and only observed a foraging ladder‐backed once, several miles outside of our survey plots. We used this dataset to create a heat map of areas most likely to contain nesting woodpeckers and then used the Point Density tool in ArcGIS version 10.3 (Environmental Systems Research Institute) to take a 500 m² fishnet sample and interpolate density values across our study location. Within areas of high woodpecker density, we placed 12.1 km^2^ survey plots in which to look for woodpecker nests (Figure S1). From mid‐April to late May 2019, we visited each plot four times using a modified spot mapping technique to record behaviors, including drumming, calling, excavation, foraging, and any mating activities to locate their nests (Bibby et al., 2000; Martin & Geupel, 1993). After locating a woodpecker nest, we placed circular 150 m^2^ grids centered around each woodpecker nest and searched the grid, focusing on trees of diameter greater than 15 cm, in bisecting transects 20 m apart every 3–5 days between April and July 2019 to locate active secondary cavity‐nesting birds (Rodewald et al., 2005). Every cavity, whether occupied or not, that could reasonably be considered suitable for nesting by any of our cavity‐nesting focal species (based on known cavity metrics) (Cardiff and Dittmann, 2002; Patten and Smith‐Pattrn, 2020; Skutch, 1969) was recorded and revisited on subsequent visits to the plot. We monitored every established nest or nesting attempt by both woodpecker and secondary cavity nesters by visiting them every 2–4 days until the nest either fledged or failed. We considered nests successful if ≥1 fledgling was observed outside the nest, which corresponds with the average success rate (1–4 fledglings) for this species (Skutch, 1969).

Specifically, we checked cavities using a telescoping blue tooth camera. While many studies on woodpeckers are limited by the difficulties in accessing cavities sometimes 40 feet in the air, the average height of cavities in our study was about 1.8 m (±0.2 m), corresponding to the short nature of the honey mesquite in the area due to low rainfall. This, combined with the efforts of a dedicated field crew, made it possible to follow the establishment and subsequent success or failure of the many nest within our study. All nests were easily accessed with either a small step stool or a short climb into the tree.

**FIGURE 2:**
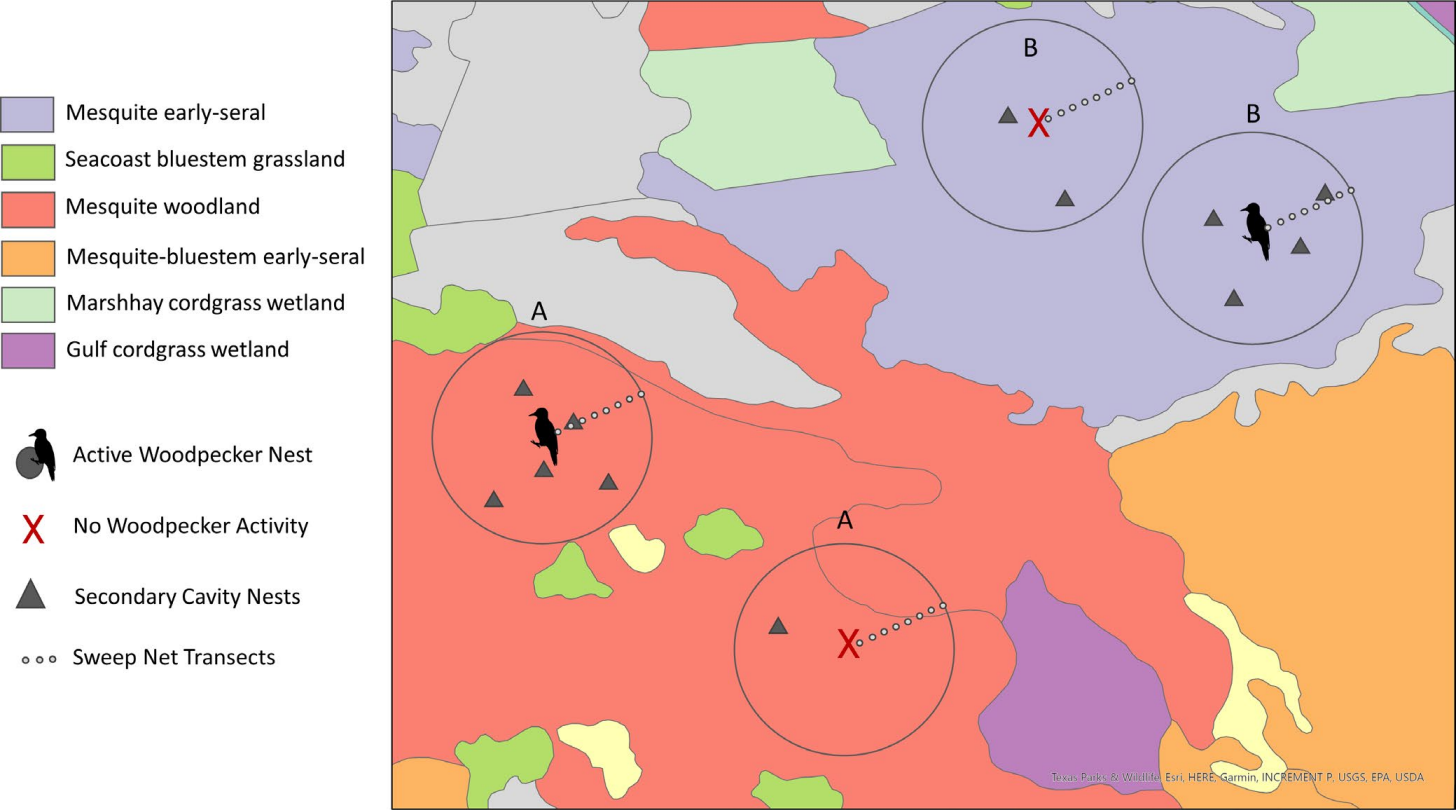
Example of nest and insect surveying plots on the San Antonio Viejo Ranch, East Foundation. After locating an active woodpecker nest, we placed a complementary location at least 300 m away that had the same vegetation association, but no woodpecker activity. Within these sites, we searched for and monitored secondary cavity‐nesting birds and conducted insect sampling with sweep net transects. Vegetation classification determined through East Foundation's hierarchical vegetation classification system, defined by the dominant and subdominant vegetation species

To determine if proximity to woodpecker foraging areas/active nests correlated with the success of secondary cavity nesters, we placed a complementary grid at least 300 m away from occupied sites that had the same vegetation association but no observed woodpecker activity (e.g., sightings, calling, drilling). Within these grids, we searched for and monitored the four most common secondary cavity nesters in the same way: black‐crested titmouse, ash‐throated flycatcher, brown‐crested flycatcher, and Bewick's wren (Baumgardt et al., 2019) (Figure 2). We recorded and monitored all cavities, regardless of the establishment of a nest or not.

We classified vegetation types with the East Foundation's hierarchical vegetation classification system, using association defined by the dominant and subdominant vegetation species (Snelgrove et al., 2013). These were created as part of a larger project by the East Foundation to classify the vegetation into a hierarchical system during the months of August‐September in 2011 and April‐June in 2012. Vegetation types were classified using a mix of satellite imagery via ArcGIS and on the ground vegetation identification. An association was based on the dominant and subdominant vegetation species. For example, an area dominated by mesquite with huisache (*Acacia farnesiana*) as the second‐most abundant species would be classified as a mesquite‐huisache association.

After a nest fledged or failed, we measured cavity metrics that have historically been predictors of nesting success. Using a flexible 5 m measuring tape, we measured the height of the cavity from the center of the cavity opening to the base of the tree (height), the diameter of the trunk or limb that the cavity was located in at the location of the cavity (diameter), diameter of the cavity opening, measured as the longest vertical distance on the face of the cavity (opening), the depth of the cavity, measured as the distance from the lowest opening of the cavity to the bottom of the cavity (depth), and decay ranking (decay), where a rank of one indicated a live tree and rank seven indicated a dead tree with no branches, no bark, and soft stem (Berl et al., 2015; Cockle et al., 2011; Dobkin et al., 1995). We also took these measurements for all cavities located during the study, whether a nest was initiated or not.

### 2.3 Foraging distances and insect sampling

The Golden‐fronted woodpecker is omnivorous; however, we focused on the insect portion of its diet to determine if woodpeckers were selecting nesting and foraging sites that corresponded with biomass of both their insect food and that of subsequent secondary cavity nesters. Using central place foraging theory (Rosenberg & McKelvey, 1999) to calculate average foraging distances, we revisited every active nest (*n* = 55) 20 times during the season and followed the male (females typically brooded chicks during the day) on foraging bouts while recording movements with a handheld GPS. We defined foraging bouts as the male leaving and returning to the nesting cavity after at least one foraging attempt and averaged the furthest distance traveled on each foraging bout, per male.

We subset sites by vegetation type (determined via the East Foundation's vegetation association) and whether or not woodpeckers were active or not at the location (Figure 2). Then, we quantified the availability of insects commonly eaten by woodpeckers and secondary cavity nesters within those sites: Hymenoptera (bees, ants, wasps), Hemiptera (true bugs), Diptera (flies), Phasmatodea (stick insects), Orthoptera (grasshoppers), and Coleoptera (beetles) (Capinera, 2011; Grubb and Pravosudov, 1994; Miles, 1990). We quantified insects with sweep nets in an array of 11 locations on a transect from the center outwards at 15 m increments (Figure 2), visiting each site twice per week from May to mid‐July 2019 (7‐week period) (Doxon et al., 2011; Grootaert et al., 2010). To survey as much insect diversity as possible, we ran the sweep net over grass, forbs, brush, and the bottoms of tree canopies (>90% of woody plants on the study site were honey mesquite, which grow low to the ground, allowing easy access to canopies) (Grootaert et al., 2010). To sample insect orders that dwell on tree trunks (mainly saproxylic beetles), we ran the sweep net up four sides of each tree (or three sides if the tree had fallen) that intercepted our sampling transects. We understand that this method is not an exhaustive sampling of saproxylic beetles, as our intent was not to extensively sample beetle populations but to find overall trends in biomass. Due to budget and field constraints, we chose this consistent method that performed well when done weekly. We followed the same methods on inactive sites to compare any differences in insect availability. We sorted the insects by order, dried them using an Elite Eliminator Heater set at 55℃, and weighed them every 24 hr until their mass stabilized.

### 2.4 Statistical analyses

#### 2.4.1 Woodpecker nest success

To compare the structure of abandoned woodpecker cavities to cavities formed by decay, we used Welch's tests for each set of measurements taken on all cavities encountered, whether a nest was initiated in them or not (Field et al., 2012). We then modeled woodpecker nesting success with respect to cavity structure. A common bias in nest success studies comes from not accounting for the number of exposure days for each nest; in short, older clutches are more likely to be successful than younger clutches (Mayfield, 1961). Given that our nests were found at various stages of development, we addressed this bias by calculating daily nest survival and modeling via logistic exposure (Shaffer, 2004; Hazler, 2004). We created models in R version 4.0.4 (2021‐02‐15) (R Core Team, 2013), with the package *lme4* (Bates et al., 2015) using the following cavity metrics: height of cavity, tree diameter, tree decay, cavity opening, and cavity depth. We considered variance inflation factors >5 as indicators of multicollinearity between variables and *z*‐scaled all continuous variables to account for varying units of measurement (O'brien, 2007).

We performed model averaging and created candidate models using the *MuMIn* package (Barton, 2009) in R to generate a model selection table (Anderson and Burnham, 2002; Field et al., 2012) and evaluated model fit using AIC adjusted for small sample sizes (AICc) (Anderson and Burnham, 2002). Since our top models had similar support (<90%), models that had ≥10% of the weight of the top model were considered candidate models for model averaging (Burnham & Anderson, 2004; Mazerolle, 2006). Using the R package *AICcmodavg* (Mazerolle & Mazerolle, 2017), we estimated the parameter coefficients through model averaging and determined which parameters were significant using α ≤ 0.05 and corresponding confidence intervals.

#### 2.4.2 Secondary cavity bird nest success

We then followed the same steps for the four secondary cavity nesters and combined observations on the ash‐throated and brown‐crested flycatchers given the similarity of their body metrics and life‐history traits, and hereafter are referred to as “flycatchers” (Cardiff & Dittmann, 2000). Using the logistic‐exposure method, we modeled the same five cavity metrics, with the addition of whether the nest was located in an abandoned woodpecker cavity or a cavity formed by decay (cavity type). As before, we used the R packages *MuMIn* and *AICcmodavg* to evaluate candidate models and average parameter coefficients per species (Table S1).

#### 2.4.3 Foraging behavior, insects, and nest success

To determine if woodpeckers were indeed foraging and nesting in areas with higher‐than‐average insect loads, we compared the most commonly eaten insects in our focal species diets between active and inactive sites: Coleoptera, Hymenoptera, Orthoptera, Hemiptera, Mantodea, Phasmatodea, and Diptera. For each site, we averaged the dried masses for each insect order over the 7‐week sampling period and used the Mann–Whitney U test to determine differences (*α* = 0.05), linking paired sites to control for differences across space. For sites occupied by a woodpecker pair, we used Spearman's Rho to test for significant correlations between each insect order and the woodpecker's average foraging distance (Field et al., 2012). Given that we predicted secondary cavity nesters would have higher success rates if nesting where woodpeckers forage, we used a chi‐square test to compare survival rates of the wren, flycatchers, and titmouse nests in radii around where woodpeckers were foraging, or not, and then again used logistic exposure to determine if any orders of insects were predictors of daily nest survival in secondary cavity‐nesting birds. We created models in R with the package *lme4* (Bates et al., 2015) for each combination of bird species daily nest survival and the biomass of collected insect orders. We considered variance inflation factors >5 as indicators of multicollinearity between variables and *z*‐scaled all continuous variables to account for varying units of measurement (O'brien, 2007).

## 3 RESULTS

### 3.1 Woodpecker nest success

**FIGURE 3:**
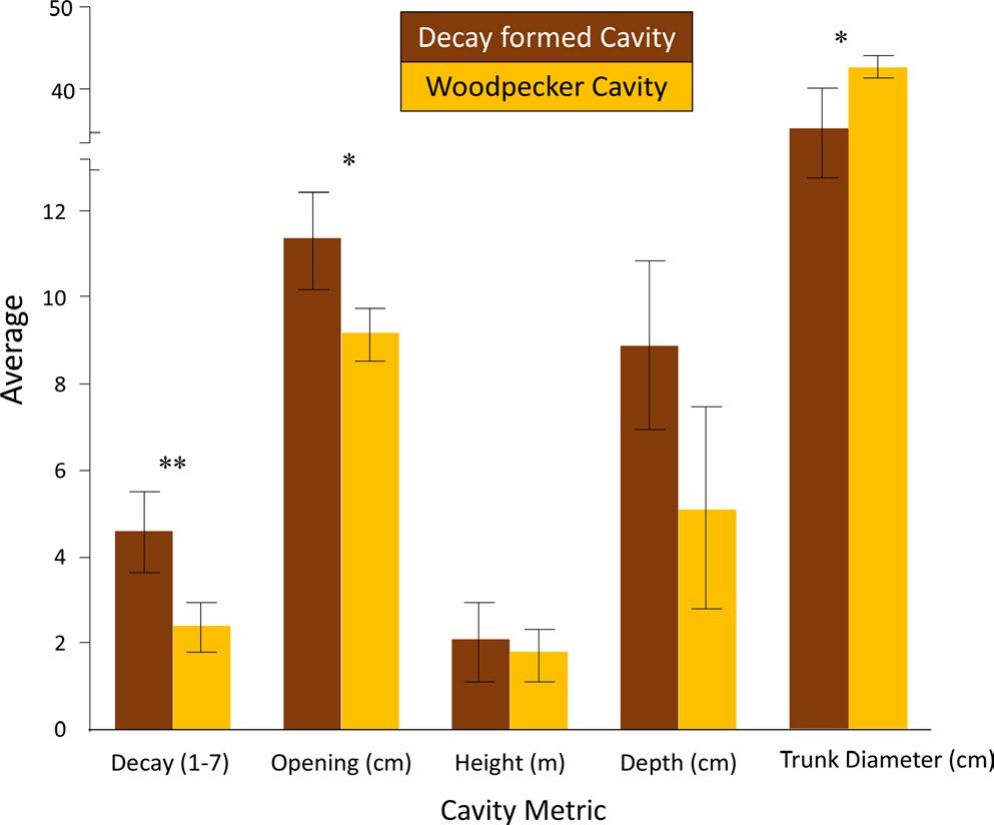
Results of Welch's *t* test comparing differences between woodpecker and decay‐formed cavities. Decay‐formed cavities *n* = 847, Woodpecker cavities *n* = 526. Error bars represent standard error. Abandoned woodpecker cavities were built in trees with significantly less decay, with smaller openings, and in trees with larger diameters, compared with decay‐formed cavities. Note the break in the *y*‐axis. Data were collected on the SAV Ranch, East Foundation during 2019

In total, we found 55 woodpecker nests, of which 40 successfully fledged ≥1 young. Across all cavities found, whether a nest had been initiated or not, abandoned woodpecker cavities (*n* = 526) were built with significantly smaller cavity entrances, and in less decayed trees with larger DBH than cavities formed by decay (*n* = 847) (Figure 3). The height and depth of the cavity were not significantly different between nest types.

After testing each variable, we found that no variance inflation factors were >5, indicating no multicollinearity; thus, all predictors were entered into the global model predicting woodpecker success (see Table S1 for candidate model selection). Model averaging suggested that woodpecker nests were less likely to be successful as decay increased (*β* = −0.62) and as the diameter of the hole increased (*β* = −1.03) (Table 1). The averaged and the top model had the same significant predictors of daily nest survival.

**TABLE 1:** Model averaged estimates with 95% confidence intervals (CI) for variables retained in the candidate model sets that predicted cavity‐nesting bird nesting success

	Model averaged *β*	*SE*	*p*	95% CI	
				Lower	Upper
Golden‐fronted woodpecker (*n* = 55)					
Decay	*−0.62*	*0.27*	*.012*	*−1.15*	*−0.09*
*Trunk diameter*	*0.02*	*0.12*	*.43*	−0.21	0.25
Diameter of opening	*−1.03*	*0.41*	*.008*	*−1.84*	*−0.21*
Height	0.03	0.16	.421	−0.35	0.28
Depth	<0.01	0.08	.49	−0.17	0.16
Bewick's wren (*n* = 79)					
Decay	0.018	0.07	.400	−0.12	0.15
Trunk diameter	0.39	0.31	.09	−0.20	1.00
Diameter of opening	−0.01	0.081	.43	−0.17	0.14
Height	−0.01	0.09	.456	−0.21	0.18
Depth	< 0.01	0.083	.478	−0.17	0.16
Cavity type (woodpecker cavity)	*2.34*	*0.75*	*.001*	*0.87*	*3.81*
Flycatchers (*n* = 102)					
Decay	*−0.27*	*0.121*	*.012*	*−0.51*	*−0.03*
Trunk diameter	−0.03	0.09	.36	−0.21	0.15
Diameter of opening	*−0.20*	*0.21*	*.042*	*−0.61*	*−0.15*
Height	0.01	0.08	.436	−0.14	0.17
Depth	−0.02	0.48	.391	−0.17	0.13
Cavity type (woodpecker cavity)	*2.22*	*0.61*	*<.001*	*1.02*	*3.41*
black‐crested titmouse (*n* = 39)					
Decay	*−0.34*	*0.16*	*<.001*	*−0.65*	*−0.02*
Trunk diameter	0.04	0.15	.394	−0.26	0.34
Diameter of opening	−0.01	0.12	.452	−0.24	0.21
Height	−0.05	0.29	.429	−0.63	0.52
Depth	0.02	0.12	.431	−0.22	0.27
Cavity type (woodpecker cavity)	*1.71*	*0.79*	*.022*	*0.17*	*3.26*

Note

All continuous variables used to create candidate models were z‐scaled. Decay was ranked 1 = live tree to 7 = dead, decayed tree. Cavity Type = whether the nest was located in an abandoned woodpecker cavity or a cavity formed by decay, with the base set as natural, beta values refer to woodpecker cavities. Flycatchers = combined observations of Ash‐throated and Brown‐crested flycatchers. Data were collected on the San Antonio Viejo Ranch, East Foundation in south Texas during the summer of 2019. Bootstrapping was used to obtain CI. *SE* is standard error and italicized variables are significant (*p* < .05).

Candidate models were chosen if they had an AICc weight ≥10% of the AICc weight of the top model.

### 3.2 Secondary cavity‐nesting success

**FIGURE 4:**
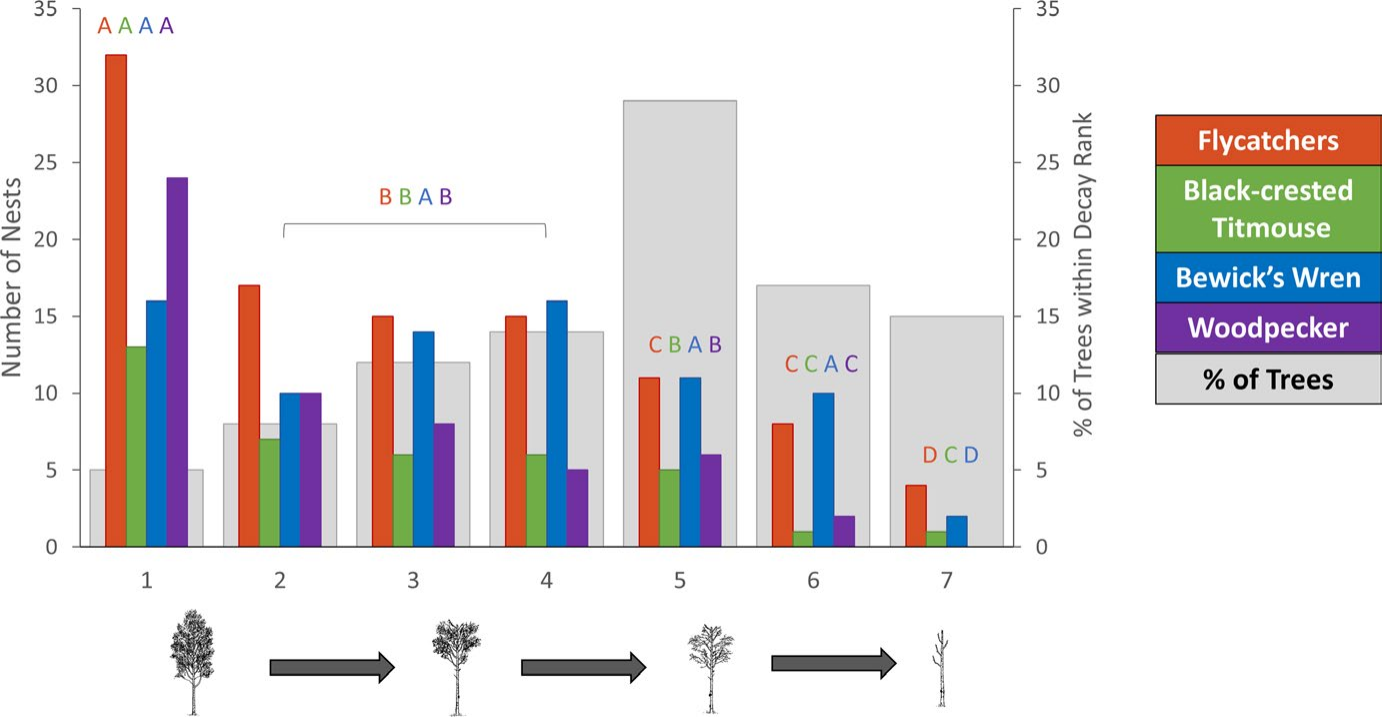
Numbers of nests found in each decay state, colored bars indicate number of nests per species, and gray bars indicate the percent of trees within the sites that fell into each decay rank. Nesting tree decay (1 = live tree, 7 = dead, decayed tree), for each cavity‐nesting bird found within the study. The data on the ash‐throated and brown‐crested flycatchers were combined (flycatchers) due to similar life‐history traits between species. Data were collected on the San Antonio Viejo Ranch, East Foundation in south Texas during the summer of 2019

In total, we found 79 wren nests, 102 flycatcher nests, and 39 titmouse nests. Excluding the wren, all species preferred to nest in trees with lower decay ranks, even though fully live trees were less common (<20%) than those of higher ranks (Figure 4).

**FIGURE 5:**
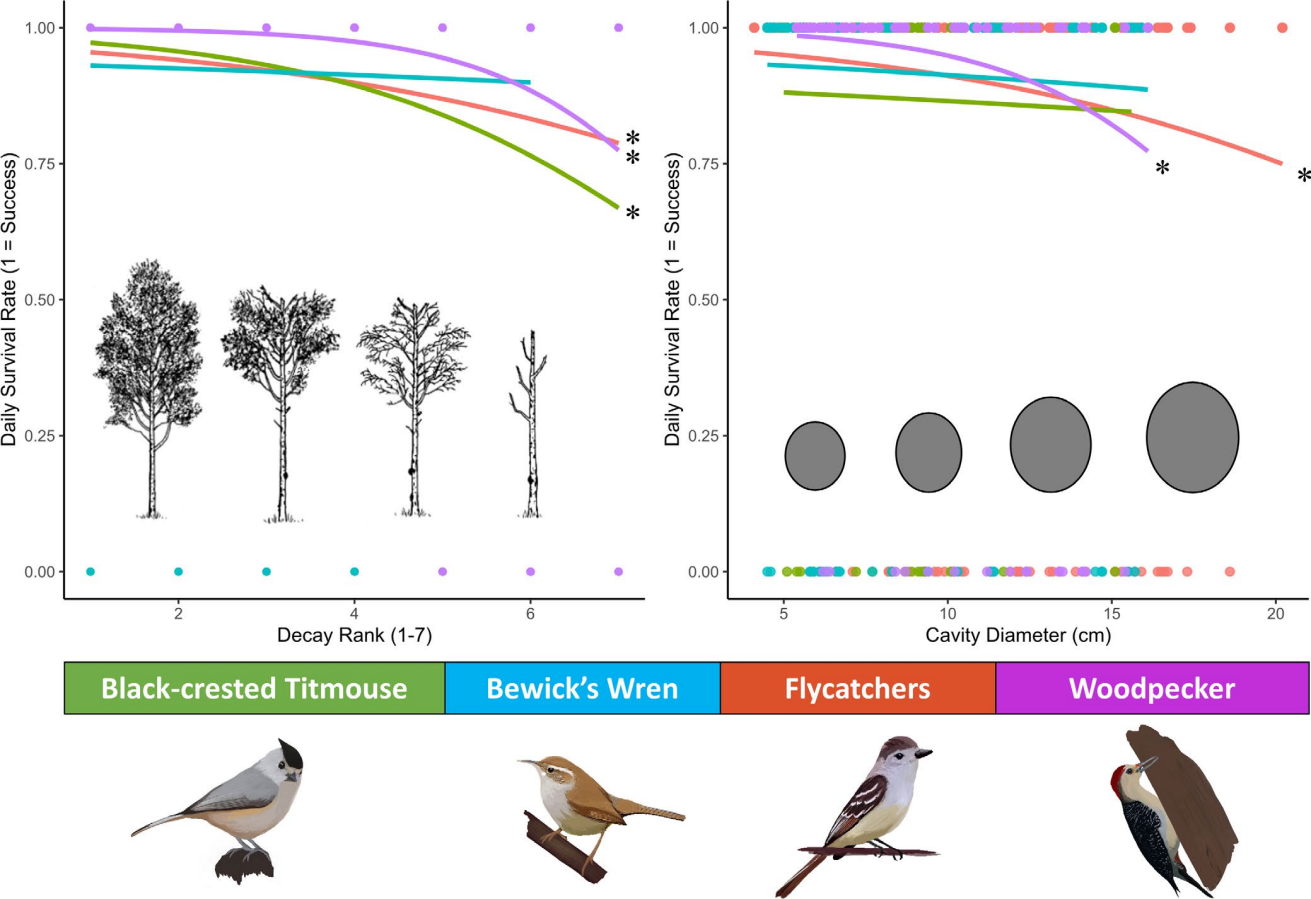
Results of logistic‐exposure model averaging looking at the daily survival rate of the woodpecker and secondary cavity‐nesting birds. The woodpecker, titmouse, and flycatchers had decreased daily nest survival as tree decay increased. Tree images as represented in surveying techniques for woodpeckers (see British Columbia Environment Ministry, 1999). Similarly, the woodpecker and the flycatchers had decreased daily nest survival as the cavity diameter increased. Asterisks indicate significance. Species drawings were done by Carmen Rosenthal Struminge

For all species of secondary cavity nesters, our model selection and averaging suggested that building in an abandoned woodpecker cavity, over a cavity formed by decay, was a strong predictor of a successful nest (Table 1). All species shared other nest predictors with the woodpecker, as both the flycatchers and the titmouse did poorer as trees became more decayed (*β* = −0.27; *β* = −0.34), and the flycatchers also did poorer as the opening of the cavity widened (*β* = −0.20) (Table 1). With every unit increase in decay, the probability of nest success dropped 0.12 for the flycatchers and 0.31 for the wren (Figure 5). Finally, for the flycatcher every unit increase in the diameter of the cavity correlated with a 0.08 decrease in the probability of the nest succeeding (Figure 5). For each species, the top model had the same significant predictors of daily survival as the averaged model.

### 3.3 Woodpecker foraging behavior and secondary cavity nester success

**FIGURE 6:**
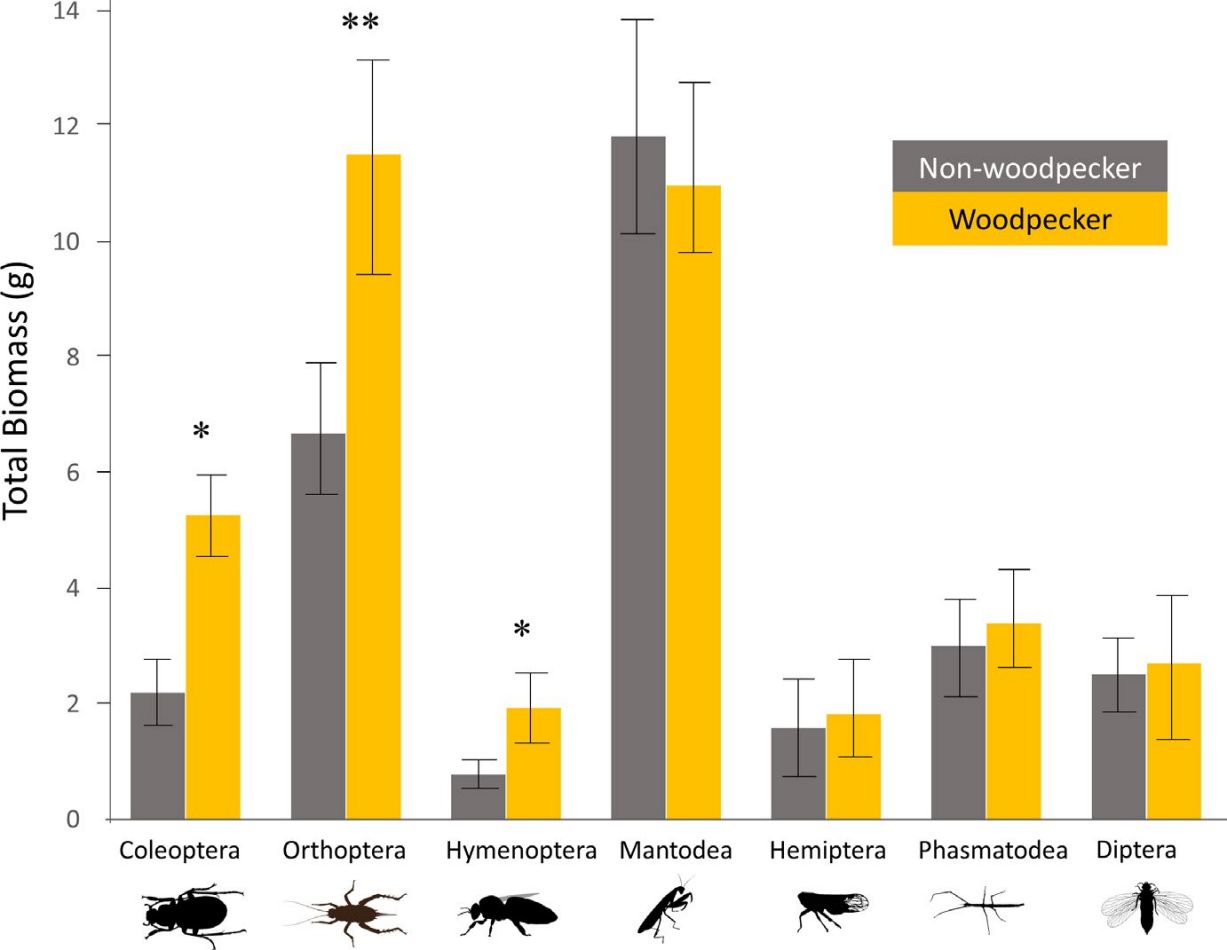
Insect samples were quantified using a sweep net and averaged across visits. Bars indicate the total biomass of each insect order on sites with and without woodpeckers; error bars represent standard error. Vegetation was controlled via East Foundation's hierarchical vegetation classification system, defined by the dominant and subdominant species

We spent approximately 50 hr recording woodpecker foraging movements; the average (+*SE*/*SD*) foraging distance per site was 102 m (±20 m). The biomasses of the insect orders Coleoptera (*n* = 358; *p* = .02), Hymenoptera (*n* = 215; *p* = <.01), and Orthoptera (*n* = 542; *p* = .03) were significantly higher on sites foraged by woodpeckers, even though Hymenoptera and Orthoptera make up a small portion of the woodpecker's diet (Figure 6). The other insect orders, Mantodea (*n* = 159), Hemiptera (*n* = 239), Phasmatodea (*n* = 102), and Diptera (*n* = 209), were not significantly different between site types.

**FIGURE 7:**
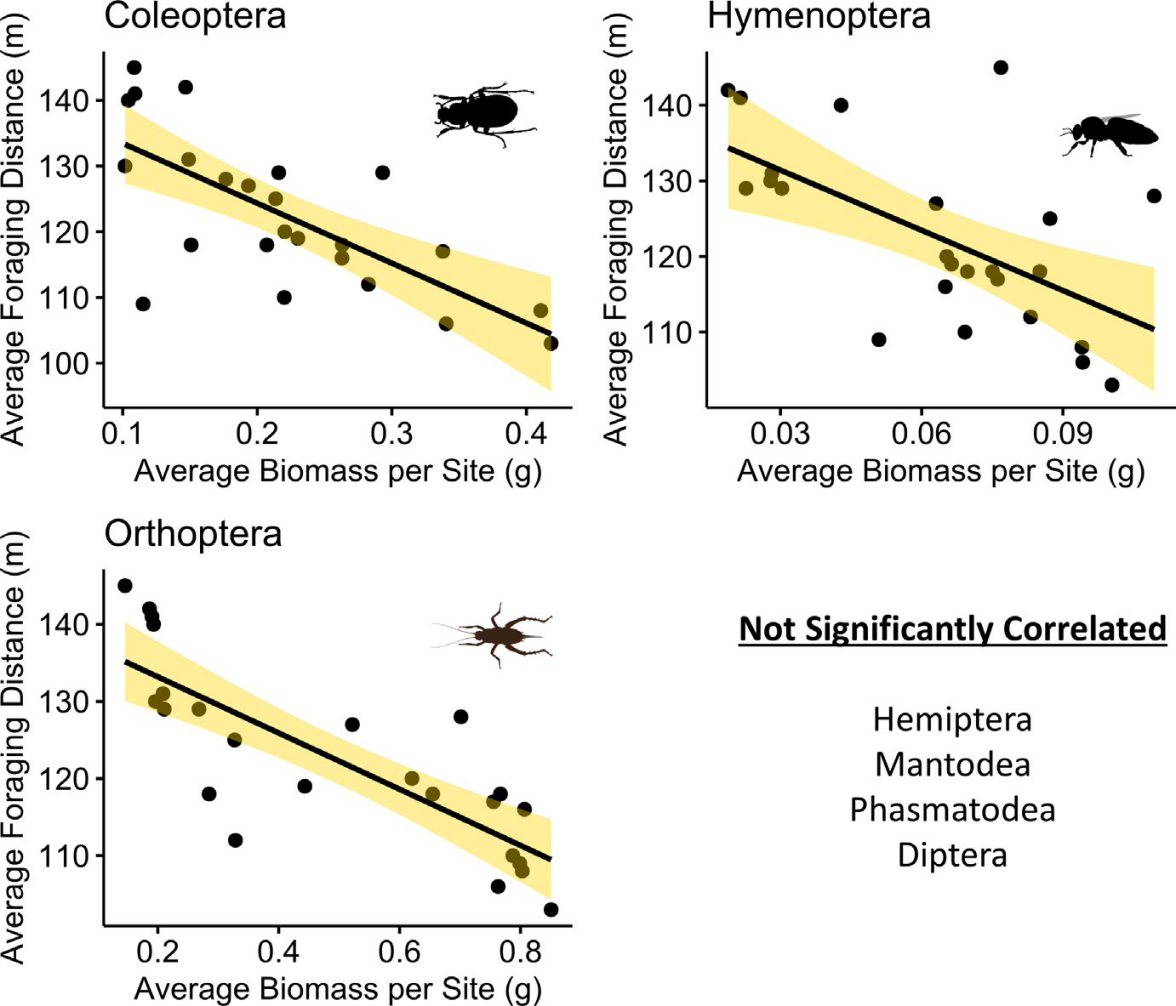
Scatter plots of Golden‐fronted woodpecker's average foraging distance, per site (m^2^) correlated with average mass (g) of significant insect orders. Shaded areas represent 95% confidence intervals. Data collected with sweep nets on the San Antonio Viejo Ranch, East Foundation in south Texas, during the summer of 2019

Similarly, the average distance a woodpecker foraged was negatively correlated with the biomass of same three orders of insects, Coleoptera (*p* < .001, rho = −0.74, *n* = 24), Orthoptera (*p* = .007, rho = −0.55, *n* = 24), and Hymenoptera (*p* = .009, rho = −0.53, *n* = 24) (see Figure 7), all other insect orders were not significantly correlated with foraging distance.

**FIGURE 8:**
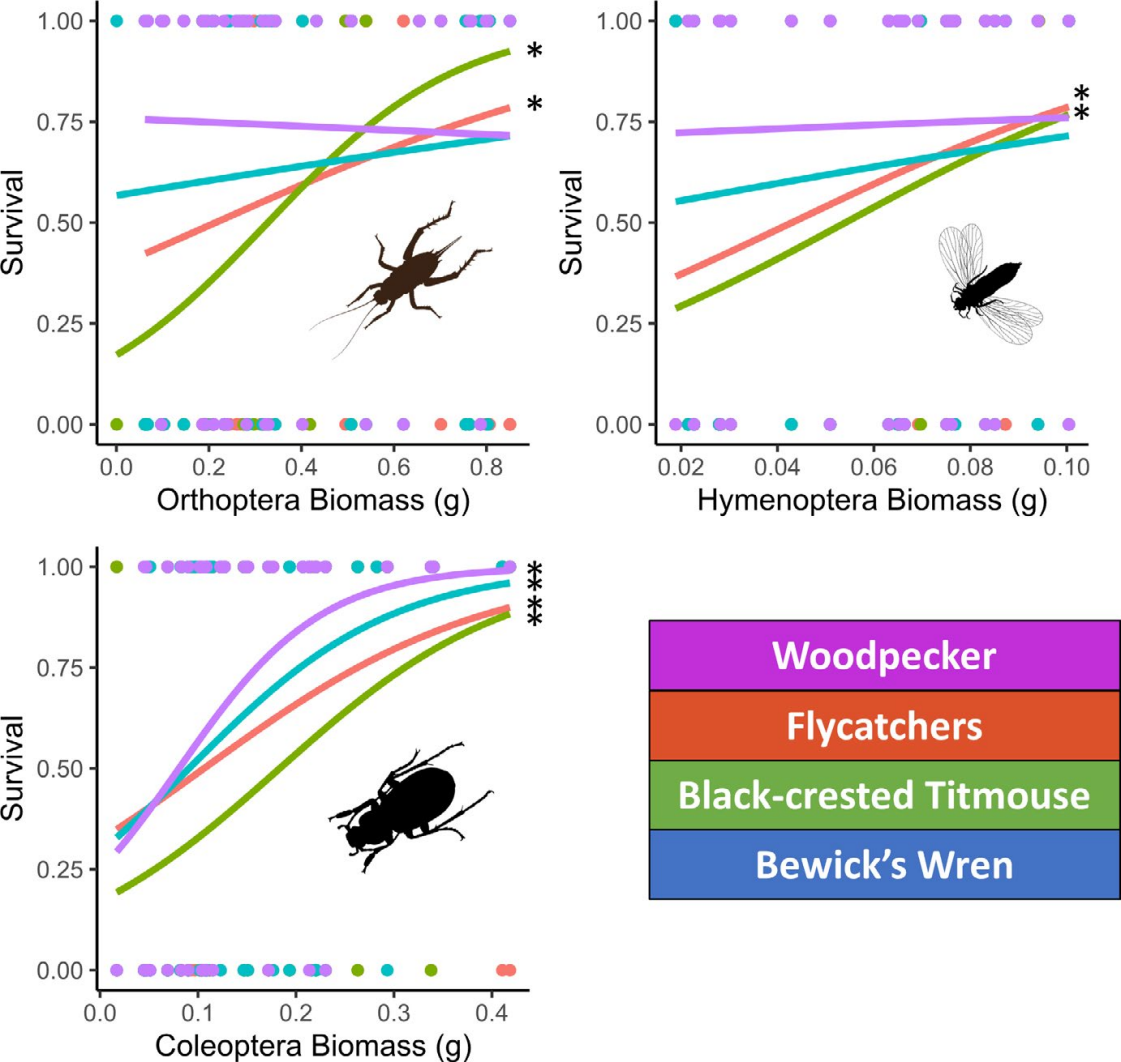
Logistic‐exposure models created for each species of cavity‐nesting bird, with each order of insect. The biomasses of Coleoptera predicted the success of all bird species, while Orthoptera and Hymenoptera were only predictive of the titmouse and flycatchers. No other sampled orders of insects were significant: Diptera, Hemiptera, Phasmatodea, Mantodea

Secondary cavity‐nesting birds had higher than expected success when nesting within an area commonly foraged in by woodpeckers, (*df* = 2; wren: *p* = .002, flycatchers: *p* < .001, titmouse: *p* = .03). Additionally, within foraging sites, the success of the woodpecker and secondary cavity‐nesting birds was correlated with the biomasses of Coleoptera, Orthoptera, and Hymenoptera, but not with any other insect orders (Figure 8). Specifically, woodpecker daily survival rate was predicted only by Coleoptera (*p* = .009); the wren was also only predicted by Coleoptera (*p* = .005), and the flycatchers and the titmouse were predicted by Coleoptera (*p* = .005; *p* = .013), Orthoptera (*p* = .27; *p* = .014), and Hymenoptera (*p* = .006; *p* = .043). (Table S2; Figure 8).

## 4 DISCUSSION

To untangle how ecosystem engineers and food resources are intertwined with local communities, we attempted to identify how the Golden‐fronted woodpecker influences secondary cavity‐nesting birds through cavity construction and potentially through nest site selection around areas with high insect availability. We found that similar cavity metrics predicted daily survival rates of woodpeckers and secondary cavity nesters alike, that all secondary cavity nesters had high nest survival rates when in an abandoned woodpecker cavity versus a cavity formed by decay, and that average woodpecker foraging distances were correlated with the biomasses of Coleoptera, Orthoptera, and Hymenoptera. Additionally, the daily survival rates of secondary cavity nesters were positively correlated with the increase of these insect orders and in turn with woodpecker foraging trends.

### 4.1 Direct engineering: interconnected nesting success

Our results suggest that woodpeckers are direct ecosystem engineers, by creating nesting cavities that increase the reproductive success of secondary cavity nesters. We predicted that secondary cavity nesters would have higher nest success in abandoned woodpecker cavities than in cavities formed by decay and found support for that hypothesis; all secondary cavity nests had higher daily survival rates when nesting within abandoned woodpecker cavities than within decay‐formed cavities. We also predicted that woodpeckers and secondary cavity nesters would have higher nest success in cavities with strong antipredation metrics: cavities built high in trees, with small openings and deep depths. We found partial support for this hypothesis; however, woodpecker and secondary cavity nesters daily nest survival were only significantly predicted by the cavity entrance diameter and the decay rate of the tree, and not cavity height or depth. The importance of these predictors also differed between species; for example, the wren's nest success was improved by being in an abandoned woodpecker cavity, but was not negatively impacted by increased tree decay. Often, we found wren nests that were built in extremely old woodpecker nests that were highly decayed and irregularly shaped. Based on previous studies, Bewick's wrens may have a high tolerance for decayed wood, as they commonly nest in completely dead trees (Taylor, 2003) and metal pipes (personal observation), and are more likely impacted by forest composition and cavity availability (Bock et al., 1992; Taylor, 2003). The flycatchers are slightly more sensitive when it comes to nesting conditions, preferring a narrower range of conditions, which would explain why their preferences mirrored those of the woodpecker (Dunning & Bowers, 1990). We were surprised by the titmouse's cavity preferences as it also has been known to nest in human structures like metal pipes (Patten and Smith‐Patten, 2020).

The diameter of the cavity was a significant predictor for both the woodpecker and the flycatchers, which matches previous cavity‐nesting literature which has suggested that small entrances are efficient antipredator defense and that rates of depredation increase with the size of the entrance hole (Mikusiński et al., 2018; Paclík et al., 2009). However, the height of the entrance did not influence nest success of any of these species, a surprising result given the antipredator benefits of high nests (Hudson & Bollinger, 2013). In fact, the majority of woodpecker nests on our site were found less than one meter off the ground. We do not have a direct explanation for this trend, but we propose two nonexclusive hypotheses: (a) all trees (>99% honey mesquite) within our sites were less than four meters tall and in general became too thin to excavate cavities in above two meters, forcing the birds to nest at low heights. However, telephone poles lining nearby roads showed evidence of woodpecker cavities that were always constructed at the very top, indicating that the birds may prefer to nest high when possible (Dennis, 1967; personal observation). (b) Tree‐climbing snakes, such as the great plains rat snake (*Pantherophis emoryi*), are the main predators of nesting birds in this region (DeGregorio et al., 2016; Davis et al., 2019) and may be able to reach any detected cavities due to the short trees. Small entrance cavities have been repeatedly shown to deter predation (Paclík et al., 2009); thus, given our results, we propose that cavity entrance rather than height is a more important predictor of cavity success in this region. Additionally, low tree height may have allowed for many of the decay‐formed cavities we monitored to shelter nonavian animals. We observed tarantulas, young snakes, and on one instance a large collection of locusts, all of which would deter a secondary cavity‐nesting bird. However, this is merely speculation and we acknowledge that we did not directly study the effects of predation, that predation pressures can fluctuate between years, and that future research is warranted to further look at the impact of environmentally stunted tree growth on reproductive decisions of cavity‐nesting birds.

Golden‐fronted woodpeckers (like many other species of woodpeckers) are known predators of smaller bird's eggs and chicks (Husak, 1995; Kujawa, 1984), and as such may act as a deterrent to secondary cavity‐nesting birds. During our study, we never saw a woodpecker attack or eat another bird's nests and at several sites observed secondary cavity nesters incubating eggs or brooding chicks in cavities within the same tree as an active woodpecker nest. Instead, we only observed the woodpeckers gleaning berries, prickly pear fruits, and small lizards, consistent with their known diet (Kujawa, 1984). On the other hand, we did opportunistically observe active predation by a rat snake on seven secondary cavity nesters (all within either highly decayed and/or decay‐formed cavities) and saw evidence of predation (shed skin on tree) on three others, highlighting the dangers of rat snakes to cavity‐nesting birds in this region. However, we did not directly measure predation and our lack of observation of woodpeckers as predators of secondary cavity nesters does not mean it never happened, and further research on the foraging ecology of Golden‐fronted woodpeckers in this region is warranted.

### 4.2 Engineering effects: resource‐driven site location

We predicted that woodpeckers would forage in areas of increased food availability and that this would correlate with an increase in secondary cavity nester success. We found support for both aspects of this hypothesis.

Similar to our findings for beetles, both Orthoptera and Hymenoptera had consistently higher biomasses on sites foraged by woodpeckers and woodpeckers took shorter foraging trips when their biomasses were high. However, we rarely observed woodpeckers consuming insects of these orders. It is possible that these insects are important components of woodpecker diet outside of our observational windows, or woodpeckers may be choosing sites based on beetle populations, and orthopteran and hymenopteran populations are simply positively correlated with beetle populations. This later hypothesis is supported by the use of beetle populations as indicators for insect species richness in other studies (Brooks et al., 2012). The positive correlation between populations of multiple insect taxa is most likely due to fine‐scale environmental trends, such as water availability and soil type that our vegetation associations were unable to distinguish (Crist et al., 2006; Zhu et al., 2014). Although biomasses of orthopterans and hymenopterans were not significant predictors of woodpecker nesting success in our models, biomasses of these insect orders were important positive predictors of secondary cavity nester success. Thus, we suggest that woodpeckers chose areas that had high foraging potential for themselves, (e.g., Coleoptera; Kujawa, 1984) which positively impacted secondary cavity nesters who may benefit from abandoned woodpecker cavities in areas with corresponding high food availability. This seems especially true for the flycatchers, whose nest success was positively predicted by all three of the correlated insect orders. Surprisingly, wren nest success was only predicted by Coleoptera biomass. However, the wren has a more flexible foraging style including hawking, exploring bark, and foraging on both ground substrate and upper canopies of trees (Miles, 1990), and may have been able to take advantage of many different food types.

### 4.3 Energetic trade‐offs in excavation

As a whole, woodpeckers within the genus *Melanerpes* excavate cavities within a mix of live and partially decayed trees, though preferentially in the latter (Bent, 1939; Kilham, 1958; Skutch, 1969). In our study area, trees with decay rank of 1–2 are less common than other ranks (<15%). However, we found 62% of woodpecker nests within trees with decay rank 1–2, and all nests were found in the main trunk of the tree. Nests of woodpeckers, wrens, and titmice also had higher success in trees with low decay ranks. All trees containing nests were honey mesquite, a dense, woody legume: Thus, when forced to excavate very dense woody species (all honey mesquite), woodpeckers still preferred the densest rank. We propose an explanation for this energy expenditure: Live trees, with higher water content, provide greater insulation against high and low temperature extremes (Grüebler et al., 2014). This insulation may improve nest success in extreme temperatures of southern Texas, where daytime temperatures frequently reached over 42.2°C. Thus, these woodpeckers may be facing an energetic trade‐off: to expend extra effort by excavating a dense, live tree, which is a better defense against temperature extremes; or risk these dangers by nesting in a decayed tree that requires less energy to excavate. Such a trend has been described before in northern flickers (*Colaptes auratus*) (Wiebe, 2017). Additionally, Golden‐fronted woodpeckers are known to reuse cavities within and across years (Bent, 1939; Skutch, 1969), a behavior we observed on several occasions and perhaps a behavior encouraged by the difficulty of cavity excavation. Similarly, the red‐cockaded woodpecker also reuses cavities and preferentially excavates live, large, trees of high density (Walters, 1991; Conner et al., 2001).

### 4.4 Future directions

In this study, we attempted to control for vegetation by using the dominant and subdominant vegetation species (vegetation associations), but more nuanced vegetation differences could exist and be influencing insect availability and nest success, for example, through predation, nest orientation, and proximity to water (Davis et al., 2019; Schaaf, 2020). Sweep netting also does not provide an exhaustive sampling of available insects, and additional sampling methods may yield further data on the relationships between insect biomass and nest success. Finally, we acknowledge that our study was conducted within the constraints of a single year and should be subjected to extra scrutiny due to fluctuating external factors such as rainfall and temperature. However, as mentioned in our study area description, the temperature and rainfall of 2019 was consistent with the 30‐year average. As such, future work should focus on multiyears influences on the role of the Golden‐fronted woodpecker as an ecosystem engineer. Other avenues to explore could include heterospecific attraction (Thomson et al., 2003). For example, are secondary cavity nesters drawn to areas occupied by woodpeckers? The use of playback calls in experimental plots could be one of many ways to test this theory (Ward & Schlossberg, 2004).

## 5 CONCLUSION

Woodpecker decisions as an ecosystem engineer impact the success of secondary cavity‐nesting birds on multiple levels. All of our secondary cavity nesters had higher success in abandoned woodpecker cavities than in cavities formed by decay, and predictors of daily nest survival were shared between taxa. Our results also suggest an additional level of interaction, with a positive correlation between the levels of insects common in these birds' diets, the daily survival rates of all cavity nesters, and woodpecker foraging trends. Additionally, the typical predictors of nest success, such as nest height, did not hold true for our study, potentially indicating a need for future research in energetic trade‐offs or woodpeckers in extreme temperatures. Overall, our results show that the influences of ecosystem engineers are complex and require multilevel approaches to understand their impact.

## CONFLICT OF INTEREST

None declared.

## AUTHOR CONTRIBUTIONS

**Faith O Hardin:** Conceptualization (lead); Data curation (lead); Formal analysis (lead); Investigation (lead); Methodology (lead); Project administration (lead); Resources (equal); Writing‐original draft (lead); Writing‐review & editing (equal). **Samantha Leivers:** Conceptualization (supporting); Data curation (supporting); Investigation (supporting); Writing‐original draft (supporting); Writing‐review & editing (equal). **Jacquelyn Grace:** Methodology (supporting); Visualization (supporting); Writing‐original draft (supporting); Writing‐review & editing (equal). **Zachary B Hancock:** Formal analysis (supporting); Methodology (supporting); Visualization (supporting); Writing‐review & editing (supporting). **Brian L Pierce:** Formal analysis (supporting); Investigation (supporting); Methodology (equal); Writing‐review & editing (supporting). **Tyler Campbell:** Funding acquisition (lead); Methodology (supporting); Resources (lead); Writing‐review & editing (supporting). **Michael L Morrison:** Conceptualization (supporting); Funding acquisition (equal); Project administration (supporting); Resources (supporting); Supervision (supporting); Writing‐original draft (supporting); Writing‐review & editing (supporting).

## Supporting information

App S1

## Data Availability

Data and analytical code are available on Figshare: 10.6084/m9.figshare.14368382 and 10.6084/m9.figshare.14368304.
