# Supplementary material for: Secondhand homes: The multilayered influence of woodpeckers as ecosystem engineers
Source: Ecol Evol. 2021 Jul 22;11(16):11425–39. doi: 10.1002/ece3.7932 (PMC8366857; doi:10.1002/ece3.7932)
Supplement: Supplementary file 1 — App S1 [file ECE3-11-11425-s001.docx]

**Supplementary**

Variables used throughout manuscript and supplementary materials:

- Height = height of the nest measured from the center of the cavity opening to the base of tree
- Trunk Diameter = diameter at breast height of the tree
- Opening = diameter (top to bottom) of the cavity opening
- Depth = depth of the cavity
- Origin = Whether the cavity was formed by decay or created by a woodpecker. “+” indicates excavated by a woodpecker.
- Decay = decay ranking, where a rank of one indicated a live tree and rank seven indicated a dead tree with no branches, bark, and soft stem

**Supplementary Table 1: Candidate Model Sets: <2 Delta**

| *Bewick's Wren* | | **Decay** | **Origin** | **Trunk Diameter** | **Depth** | **Height** | **Opening** | **DF** | **logLik** | **AICc** | **delta** | **weight** |
| --- | --- | --- | --- | --- | --- | --- | --- | --- | --- | --- | --- | --- |
|  | M1 | NA | + | NA | NA | NA | NA | 3 | -79.14 | 164.37 | 0.00 | 0.17 |
|  | M2 | 0.11 | + | 0.55 | NA | NA | NA | 4 | -78.83 | 165.80 | 1.43 | 0.08 |
|  | M3 | NA | + | NA | NA | NA | NA | 2 | -80.96 | 165.96 | 1.59 | 0.08 |
|  | M4 | NA | + | 0.43 | NA | NA | -0.11 | 4 | -79.00 | 166.13 | 1.76 | 0.07 |
|  | M5 | NA | + | 0.45 | NA | -0.09 | NA | 4 | -79.09 | 166.32 | 1.95 | 0.06 |
|  |  |  |  |  |  |  |  |  |  |  |  |  |
| *Flycatchers* | |  |  |  |  |  |  |  |  |  |  |  |
|  | M1 | -0.25 | + | NA | NA | NA | -0.29 | 4 | -118.5 | 245.10 | 0.00 | 0.15 |
|  | M2 | -0.29 | + | NA | NA | NA | NA | 3 | -119.9 | 245.86 | 0.76 | 0.10 |
|  | M3 | -0.26 | + | NA | -0.14 | NA | -0.34 | 5 | -118.1 | 246.34 | 1.24 | 0.08 |
|  | M4 | -0.27 | + | -0.12 | NA | NA | -0.27 | 5 | -118.2 | 246.46 | 1.36 | 0.08 |
|  | M5 | -0.24 | + | NA | NA | 0.11 | -0.27 | 5 | -118.3 | 246.83 | 1.73 | 0.06 |
|  | M6 | -0.31 | + | -0.14 | NA | NA | NA | 4 | -119.4 | 246.89 | 1.79 | 0.06 |
|  |  |  |  |  |  |  |  |  |  |  |  |  |
| *Black-crested Titmouse* | | |  |  |  |  |  |  |  |  |  |  |
|  | M1 | -0.33 | + | NA | NA | NA | NA | 3 | -46.28 | 98.73 | 0.00 | 0.19 |
|  | M2 | -0.35 | + | 0.20 | NA | NA | NA | 4 | -46.02 | 100.32 | 1.59 | 0.09 |
|  | M3 | -0.34 | + | NA | 0.13 | NA | NA | 4 | -46.18 | 100.64 | 1.63 | 0.07 |
|  | M4 | -0.32 | + | NA | NA | NA | -0.09 | 4 | -46.23 | 100.74 | 1.75 | 0.07 |
|  | M5 | -0.34 | + | NA | NA | -0.04 | NA | 4 | -46.27 | 100.83 | 1.84 | 0.07 |
|  | M6 | NA | + | NA | NA | NA | NA | 2 | -48.78 | 101.65 | 1.90 | 0.04 |
|  | M7 | -0.33 | + | 0.22 | NA | NA | -0.12 | 5 | -45.93 | 102.28 | 1.98 | 0.03 |
|  |  |  |  |  |  |  |  |  |  |  |  |  |
|  |  |  |  |  |  |  |  |  |  |  |  |  |
| *Golden-fronted Woodpecker* | | | **Decay** | **Trunk Diameter** | **Depth** | **Height** | **Opening** | **DF** | **loglik** | **AICc** | **delta** | **weight** |
|  |  | M1 | -0.62 | NA | NA | NA | -1.01 | 3.00 | -53.38 | 112.84 | 0.00 | 0.31 |
|  |  | M2 | -0.63 | NA | NA | -0.17 | -1.08 | 4.00 | -53.25 | 114.64 | 1.80 | 0.13 |
|  |  | M3 | -0.60 | 0.11 | NA | NA | -1.01 | 4.00 | -53.29 | 114.70 | 1.86 | 0.12 |
|  |  | M4 | -0.63 | NA | -0.02 | NA | -1.01 | 4.00 | -53.38 | 114.89 | 1.96 | 0.11 |

|  | | | | | |  |
| --- | --- | --- | --- | --- | --- | --- |
|  |  |  |  |  |  |  |
|  |  | **Estimate** | **SE** | ***z* value** | **P** |  |
| **Coleoptera** | |  |  |  |  |  |
|  | Flycatchers* | 7.01 | 2.49 | 2.81 | 0.005 |  |
|  | Wren* | 9.69 | 3.48 | 2.79 | 0.005 |  |
|  | Titmouse* | 8.61 | 3.46 | 2.48 | 0.013 |  |
|  |  |  |  |  |  |  |
| **Orthoptera** | |  |  |  |  |  |
|  | Flycatcher* | 2.04 | 0.92 | 2.21 | 0.027 |  |
|  | Wren | 0.76 | 0.99 | 0.76 | 0.450 |  |
|  | Titmouse* | 4.80 | 1.96 | 2.46 | 0.014 |  |
|  |  |  |  |  |  |  |
| **Hymenoptera** | |  |  |  |  |  |
|  | Flycatcher* | 22.74 | 8.29 | 2.74 | 0.006 |  |
|  | Wren | 8.74 | 9.45 | 0.92 | 0.360 |  |
|  | Titmouse* | 25.83 | 13.90 | 1.86 | 0.063 |  |
|  | | | | | |  |

**Supplementary Table 2:**Full logistic exposure model results of cavity nesting birds’ daily nest survival in relation to insect biomass. Asterisk denote significance.

**Supp Figs.**


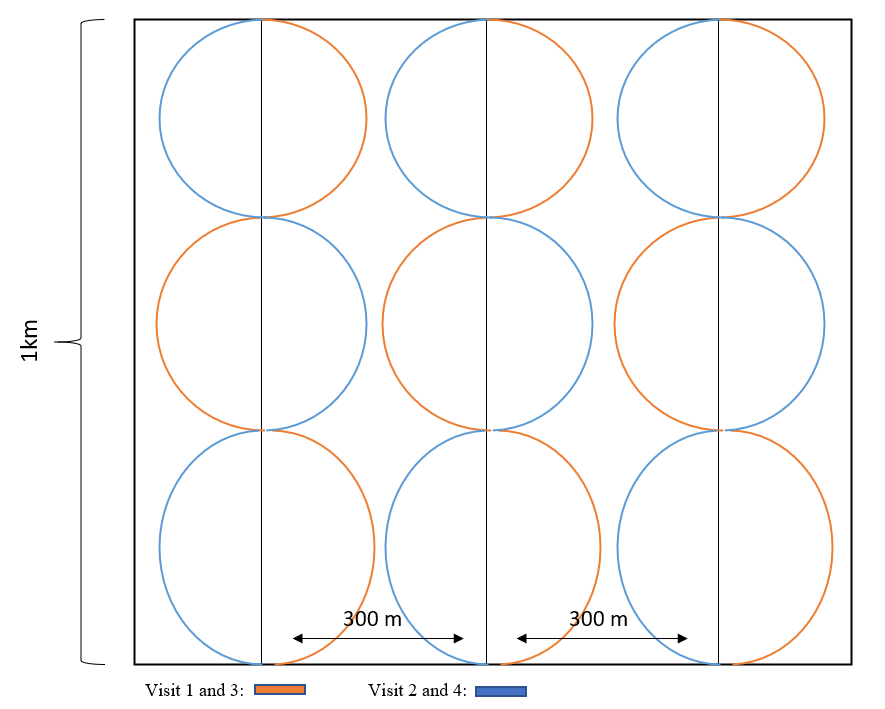


**Sup Fig. 1:** Visual of walking transects used to detect and locate Golden-fronted woodpecker nests throughout the 12, 1 km plots placed on the San Antonio Viejo Ranch, East Foundation, during spring and summer 2019. Orange path was followed during visits 1 and 3, blue path was followed for visits 2 and 4.
